# Supplementary material for: The DEXACELL trial—a protocol for a pragmatic, multicentre, double-blind, placebo-controlled, randomised, parallel group, phase 3 superiority trial to assess the effectiveness and cost-effectiveness of DEXAmethasone as an adjunctive therapy for the management of CELLulitis in adults presenting to urgent secondary care in the UK
Source: BMJ Open. 2025 Oct 29;15(10):e109953. doi: 10.1136/bmjopen-2025-109953 (PMC12574402; doi:10.1136/bmjopen-2025-109953)
Supplement: online supplemental file 1 [file bmjopen-15-10-s001.pdf]

<Trust Logo>

The DEXACELL Trial

Participant Consent Form

Participant ID: ______________________

<u>If the participant is unable to provide written consent (e.g. due to cellulitis affecting the hand) and provides verbal consent only:</u> please tick this box. In this case, an **independent** witness should enter their own initials in each of the statement boxes (e.g. a clinician on duty not involved in the trial) and countersign this form. ☐

CONSENT STATEMENTS	PARTICIPANT INITIALS
1. I confirm that I have received the Participant Information Sheet dated .... (version...........) for the above study. I have had the opportunity to consider the information, ask questions and have had these answered satisfactorily.	Please Initial
2. I understand that my participation is voluntary and I can stop taking part at any time without giving any reason and without my medical care or legal rights being affected.	Please Initial
3. I agree that if I withdraw from the study any information already collected about me will be retained and I may still be contacted if any new significant information becomes available in regard to safety and the treatment I have received in the trial.	Please Initial
4. I understand that relevant sections of my medical notes and data collected during the study may be looked at by individuals from the University of Exeter, University of Bristol, University of Cardiff, North Bristol NHS Trust, regulatory authorities or the recruiting NHS Trust, where it is relevant to my taking part in this research. I give permission for these individuals to have access to my records.	Please Initial
5. I agree for a copy of my consent form, including my name, to be sent to and stored by the Clinical Trials Unit at the University of Exeter for review.	Please Initial
6. I agree to my General Practitioner (GP) being informed about my participation in the study.	Please Initial
7. I understand that the information held and maintained by the University of Exeter, North Bristol NHS Trust and recruiting NHS Trust may be used to help contact me in relation to the trial.	Please Initial
8. I agree for my mobile number to be shared with the SMS service provider (based in the United States) who will send the follow-up text messages.	Please Initial
9. I agree to the processing of my personal information for the purposes explained to me. I understand that such information will be handled in accordance with the terms of the UK General Data Protection Regulation (GDPR).	Please Initial
10. I understand that the information collected about me may be used to support other research in the future and may be shared anonymously with other researchers.	Please Initial
11. I agree to take part in the DEXACELL trial.	Please Initial
12. **<u>Participants of child-bearing potential only:</u>** I agree to use at least one form of effective contraception from the time consent is signed until 3 months after my final dose of the study drug.	Please Initial

OPTIONAL **CONSENT ITEMS** - *Please tick yes or no and initial*	**YES**	**NO**	**PARTICIPANT INITIALS**
1. I agree to my information (date of birth and NHS number/CHI number) being shared with the relevant health service - NHS England, NHS Wales Informatics Service or Information Services Division (ISD) Scotland depending on the country I live in. This will be to obtain information about my health status for future research, as described in the participant information sheet.			Please Initial
2. I agree to be contacted about ethically approved research studies for which I may be suitable in future. I understand that agreeing to be contacted does not oblige me to participate in any further studies.			Please Initial
3. I would like to receive newsletters about progress of the study and agree to my contact details being stored by the University of Exeter for this purpose.			Please Initial
4. I would like to receive a summary of the study results once it is finished, including confirmation of the treatment I was allocated to, and agree to my contact details being stored by the University of Exeter for this purpose.			Please Initial

**CONTACT PREFERENCE**	**EMAIL**	**POST**
If you opted to receive the study results or newsletters above, please indicate in which format you would prefer to receive these.		

		_ _ - _ _ - 2 0 _ _
**Participant name**	**Participant signature**	**Date signed (dd/mm/yyyy)**
		_ _ - _ _ - 2 0 _ _
**Name of researcher seeking consent**	**Researcher signature**	**Date signed (dd/mm/yyyy)**

<u>Only for completion if participant consented verbally:</u>

The patient is unable to sign/complete this form. As a witness, I confirm that all the information about the study was given, and the patient gave their fully informed consent to take part as indicated in this form.

		_ _ - _ _ - 2 0 _ _
**Witness name**	**Witness signature**	**Date signed (dd/mm/yyyy)**

Note to consenting researcher: Original in the site file, copy to the participant, copy scanned or filed into the medical notes and unredacted copy provided to Exeter Clinical Trials Unit via REDCap upload.

This study is funded by the National Institute for Health Research (NIHR) Health Technology Assessment (HTA) Programme (NIHR153216). The views expressed are those of the author(s) and not necessarily those of the NIHR or the Department of Health and Social Care.

FUNDED BY NIHR | National Institute for Health and Care Research
