# Supplementary material for: The DEXACELL trial—a protocol for a pragmatic, multicentre, double-blind, placebo-controlled, randomised, parallel group, phase 3 superiority trial to assess the effectiveness and cost-effectiveness of DEXAmethasone as an adjunctive therapy for the management of CELLulitis in adults presenting to urgent secondary care in the UK
Source: BMJ Open. 2025 Oct 29;15(10):e109953. doi: 10.1136/bmjopen-2025-109953 (PMC12574402; doi:10.1136/bmjopen-2025-109953)
Supplement: online supplemental file 2 [file bmjopen-15-10-s002.docx]

# Supplementary File S2:

# Numerical rating scale of pain wording

Participants will receive the following wording either via SMS or spoken to them by a member of the trial team if SMS if data is being collected in person or on the phone.

“Please rate the level of pain you are currently experiencing due to your cellulitis. The scale is from 0-10. Where 0 means no pain at all and 10 means the worst pain you can imagine.”
