# Supplementary material for: The DEXACELL trial—a protocol for a pragmatic, multicentre, double-blind, placebo-controlled, randomised, parallel group, phase 3 superiority trial to assess the effectiveness and cost-effectiveness of DEXAmethasone as an adjunctive therapy for the management of CELLulitis in adults presenting to urgent secondary care in the UK
Source: BMJ Open. 2025 Oct 29;15(10):e109953. doi: 10.1136/bmjopen-2025-109953 (PMC12574402; doi:10.1136/bmjopen-2025-109953)
Supplement: online supplemental file 3 [file bmjopen-15-10-s003.docx]

# Supplementary File S3:

# Risk assessment of short term side effects of dexamethasone

**Hyperglycaemia:** We are including people with diabetes in this trial, due to their increased risk of cellulitis. However, dexamethasone can produce prolonged hyperglycaemia in people with diabetes, requiring insulin or other medications to control it. The absolute risk of severe hyperglycaemia with dexamethasone use appears low from recent trial data, however, given the potential seriousness of this risk, site-specific training and work instructions will be provided to sites covering hyperglycaemia, sick day rules, and advice on diabetes management. Participants with diabetes will be given additional patient information sheets to ensure they are fully informed prior to consent. These incorporate Diabetes UK guidance on sick day rules and how to manage high sugar levels. Cases of severe hyperglycaemia (ketoacidosis, hyperglycaemic hyperosmolar state or hyperglycaemia requiring new use of insulin) will be reported as serious adverse events in this trial and monitored by the trial oversight committees.

**Gastrointestinal (GI) toxicity/bleeding:** The risk of GI bleeds is relatively low with corticosteroids; however, this risk may increase with other factors including peptic ulcer disease, use of non-steroidal anti-inflammatory drugs (NSAIDs) and older age. People with active gastric or duodenal ulceration will not be eligible for this trial due to this increased risk. As NSAIDs may be prescribed as part of standard practice to manage the pain of cellulitis and given the short course of corticosteroids being used and the fact that NSAIDs are not listed as a contraindication in the dexamethasone SmPC, co-administration is allowed for this trial. To mitigate any risk, sites are provided with a working instruction on assessing the risk of GI bleeding in patients. Clinicians are advised to consider prescribing a proton pump inhibitor if felt to be clinically indicated, based on the participant’s risk factors and recent NSAID usage. Cases of gastrointestinal bleeding will be reported as serious adverse events in this trial and monitored by the trial oversight committees.

**Psychosis:** Psychosis is a recognised and serious complication of corticosteroid treatment, but it is related to dose and length of corticosteroid treatment.^1^ It is therefore likely to be a very rare event in this trial given the short course and low dose of dexamethasone being used. Cases of psychosis will be reported as serious adverse events in this trial and monitored by the trial oversight committees.

1. Warrington, T. P. & Bostwick, J. M. Psychiatric Adverse Effects of Corticosteroids. *Mayo Clin. Proc.* **81**, 1361–1367 (2006).
