# Supplementary material for: The DEXACELL trial—a protocol for a pragmatic, multicentre, double-blind, placebo-controlled, randomised, parallel group, phase 3 superiority trial to assess the effectiveness and cost-effectiveness of DEXAmethasone as an adjunctive therapy for the management of CELLulitis in adults presenting to urgent secondary care in the UK
Source: BMJ Open. 2025 Oct 29;15(10):e109953. doi: 10.1136/bmjopen-2025-109953 (PMC12574402; doi:10.1136/bmjopen-2025-109953)
Supplement: Supplementary file 4 [file bmjopen-2025-109953.reviewer_comments.pdf]

PEER REVIEW HISTORY

BMJ Open publishes all reviews undertaken for accepted manuscripts. Reviewers are asked to complete a checklist review form and are provided with free text boxes to elaborate on their assessment. These free text comments are reproduced below.

ARTICLE DETAILS

Title (Provisional)

The DEXACELL Trial – a protocol for a pragmatic, multicentre, double-blind, placebo-controlled, randomised, parallel group, phase 3 superiority trial to assess the effectiveness and cost-effectiveness of DEXAmethasone as an adjunctive therapy for the management of CELLulitis in adults presenting to urgent secondary care in the UK

Authors

Joyce, Katherine; Lear, Rebecca; Hamilton, Fergus W.; Arnold, David; Chaudhuri, Ella; Connors, James; Cook, Heather; Creanor, Siobhan; Dawe, Phoebe; Goodwin, Elizabeth; Hawton, Annie; Hayward, Christopher; Lasserson, Daniel S; Ridd, Matthew J; Rowe, David; Shipley, Debbie; Taylor, Hazel; Wainman, Hannah E; Williams, O Martin; Carlton, Edward

---

This article was not externally reviewed at BMJ Open. Protocols that have been independently assessed prior to submission to BMJ Open are usually fast-tracked to publication on the grounds that further substantial changes will not be possible. This independent assessment will usually be external, independent review for both a substantial grant award from a non-commercial or government funder and ethics approval.
